# Case Report: Distal tibial physeal fracture secondary to septic physitis and osteomyelitis in a Boxer puppy

**DOI:** 10.3389/fvets.2026.1881764

**Published:** 2026-08-26

**Authors:** Nisha Sidhu, Jennifer W. Cao, Steven W. Frederick, Aidan Chambers

**Affiliations:** 1 Small Animal Medicine and Surgery, BluePearl Pet Hospital, Kirkland, WA, United States; 2 Medical Affairs, Mars Veterinary Health, Vancouver, WA, United States

**Keywords:** lameness, osteomyelitis, *Pasteurella* spp., physeal fracture, Salter-Harris type I, septic physitis

## Abstract

Salter-Harris fracture secondary to septic physitis has been reported in foals but not puppies. In this case, a 19-week-old, 13.4 kg male intact Boxer puppy was presented with a 2-day history of acute non-weight-bearing lameness of the left pelvic limb, accompanied by distal limb swelling and lethargy. Orthogonal tibial radiographs were performed, and irregular widening of the distal tibial physis with severe edema of the surrounding soft tissues consistent with septic physitis were identified. Fifteen hours later, distal tibial radiographs were repeated, and a Salter-Harris type I fracture of the distal tibial physis, presumed to be pathologic in origin, was diagnosed. Surgical exploration revealed a subperiosteal abscess, which was drained and cultured. The physeal fracture was stabilized with cross-pin fixation, which was reinforced with a splint and bandage for the duration of recovery. *Pasteurella spp*. was isolated, and amoxicillin-clavulanate was prescribed for 72 days. Resolution of swelling, radiographic fracture healing, and return to normal limb function were achieved after 14 weeks postoperative, at which time the pins were removed and submitted for aerobic and anaerobic culture and susceptibility. No bacterial isolates were identified. This is the first documented case of an appendicular physeal fracture secondary to suspected septic physitis in a dog, and septic physitis should be considered a differential for puppies with spontaneous Salter-Harris fracture.

## Introduction

Osteomyelitis refers to inflammation of the bone and bone marrow and is most frequently associated with infection by pyogenic organisms (1, 2). Osteomyelitis may arise through direct inoculation of pathogens following trauma or through hematogenous spread of infectious agents (3–5). Metaphyseal osteomyelitis in juvenile animals is thought to be primarily from hematogenous infectious agents due to the termination of capillary endothelium at the primary spongiosa within the metaphysis allowing extravasation of circulating bacteria (6). When hematogenous infectious agents cause localized infection at the growth plates of juvenile bones it is referred to as septic physitis (7, 8).

Septic physitis is a rare but severe infection of the growth plates of developing bones. It is a clinically important condition in veterinary medicine that is primarily described as a manifestation of musculoskeletal sepsis in foals and calves (7–10). In foals and calves, septic physitis is associated with pathologic fracture of the physes. Though, that association has not been documented in dogs.

Fractures involving the physeal components (Salter-Harris) result in displacement and disruption of the physeal growth plates (11). They can be traumatic or atraumatic (e.g., genetic, nutritional) in origin. Regardless of etiology, they can result in stunted long bone growth with or without axial and rotational deformities of the bone, necessitating surgical correction for optimal outcomes in many cases.

While osteomyelitis has been reported as a cause of lameness and physeal disruption in dogs, an association between infectious physitis and physeal fracture has not been established (1). The objective of this case report is to describe the successful treatment of a puppy with a distal tibial Salter-Harris type I fracture suspected to be secondary to septic physitis.

## Case summary

A 19-week-old, 13.4 kg male Boxer puppy was presented to the emergency service of a veterinary referral hospital with a 2-day history of acute, non-weight bearing left pelvic limb lameness associated with swelling and lethargy (Table 1). There was no known history of trauma to the limb. On physical examination, cardiothoracic auscultation was normal, and the left popliteal lymph node was enlarged; no other peripheral lymphadenopathy was noted. The dog was febrile (40.7 °C). The left pelvic limb was painful on manipulation and edematous from mid-femur extending distally to the paw, most pronounced around the tarsus. There was no evidence of wounds or other external injuries.

**Table 1:** Care timeline for a 19-week-old Boxer puppy presented for acute left pelvic limb lameness and hock edema that was diagnosed with a distal tibial Salter-Harris fracture secondary to suspected septic physitis and was successfully treated with internal fixation and antibiotic therapy.

Progression Date	Case Progression
Day 0	•Physical examination, biochemistry, hematology, and tick serology •Radiographs of the left hock •Acute suspected septic physitis and abscessation
Day 1	•Repeat radiographs •Salter-Harris fracture •Open reduction and internal fixation •Tissue culture •Jackson-Pratt drain •Amoxicillin/clavulanic acid prescribed •Lateral splint placed
Day 4	•Drain removed •Lateral splint replaced •*Pasteurella* spp. identified •Amoxicillin/clavulanic acid continued
Day 31	•Radiographic evaluation •Physitis improvement, cross pin complications, osteogenesis •Cross pins surgically removed •Tissue culture •Lateral splint replaced
Day 37	•No organism growth •Lateral splint replaced •Amoxicillin/clavulanic acid continued for 6 more weeks
Day 45	•Coaptation removed
Day 57	•Radiographic evaluation •Progressive osteogenesis •Improved chronic osteomyelitis with no evidence of edema or abscessation

**Figure 1:**
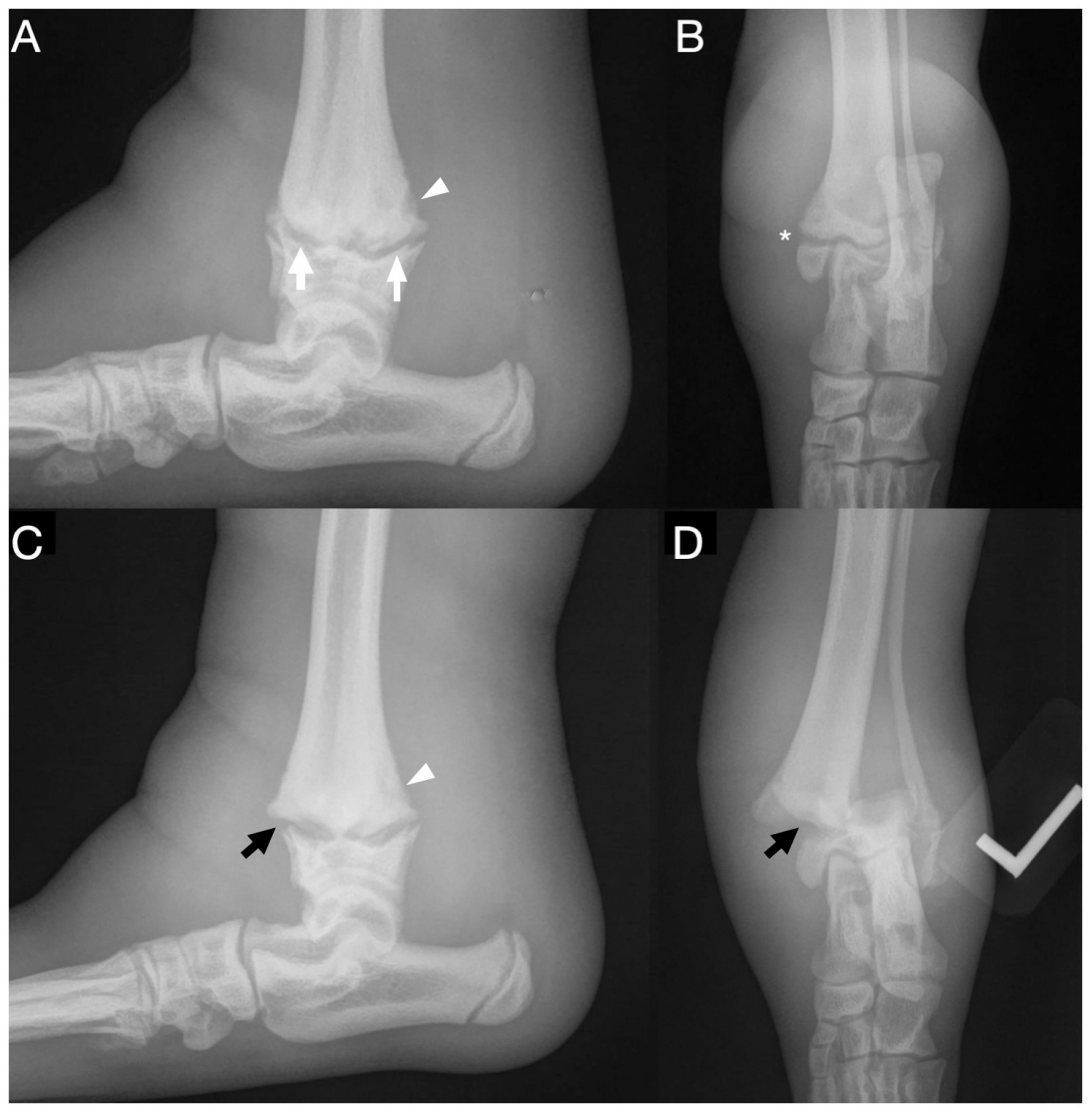
Serial lateral and craniocaudal projections of the left hock of a 19-week-old Boxer puppy taken 15 h apart while hospitalized for acute edema and lameness of the distal left pelvic limb. **(A, B)** Radiographs performed on day 0 following physical examination showed **(A)** the distal physis of the left tibia is widened (white arrows), slightly irregular, and the surrounding bone is heterogenous with irregular periosteal proliferation (white arrow points) and **(B)** severe soft tissue swelling of the distal crus, tarsus, and pes, which is most severe surrounding the distal tibial physis. There is incomplete unification of the medial malleolus of the tibia (*). **(C, D)** Radiographs performed on day 1 after 15 h of hospitalization showed **(C)** a distal tibial Salter-Harris type I fracture (black arrows) secondary to suspected septic physitis and **(D)** severe soft tissue swelling, indicating cellulitis, potentially with concurrent subcutaneous abscess formation.

On day 0 at presentation, hematology revealed mild anemia (hematocrit 32.5%, reference interval [RI] 37.3%−61.7%) and moderate neutrophilic leukocytosis (WBC 24.09 K/μL, RI 5.05–16.76 K/μL; neutrophil 18.93 K/μL, RI 2.95–11.64 K/μL). Serum biochemistry showed mildly increased alanine aminotransferase (ALT) and moderately increased alkaline phosphatase (ALP) activity (131 U/L, RI 8–75 U/L; 828 U/L, RI 46–337 U/L, respectively). Point-of-care ELISA testing (SNAP 4DX Plus, IDEXX Laboratories, Inc, Westbrook, ME, USA) was negative for *Dirofilaria immitis, Borrelia burgdorferi, Ehrlichia* spp, and *Anasplama* spp. Orthogonal radiographic projections of the left tarsus (*n* = 2) and left stifle (*n* = 2) were performed on day 0 and reviewed by a board-certified veterinary radiologist (Figures 1A, B). A widened, slightly irregular distal tibial physis with heterogeneous surrounding bone and subtle periosteal proliferation and flared medial and lateral margins of the physis were identified. Additionally, severe soft tissue swelling extended from the distal crus to the pes, most pronounced at the level of the distal tibial physis, and incomplete unification of the medial malleolus were noted. These findings were interpreted as most consistent with suspected septic physitis with severe cellulitis and edema, with reactive lymphadenopathy. Differential diagnoses included infectious or inflammatory etiologies, with immune-mediated disease, toxicity, hypersensitivity, or neoplasia considered less likely. Cellulitis, insect or bite wounds, foreign body, and arthropathy were also considered.

The dog was hospitalized for stabilization, pain control, and monitoring until a board-certified veterinary surgeon could evaluate the dog. Intravenous fluid therapy (Plasmalyte A 100 mL/kg/day), ampicillin/sulbactam (30 mg/kg IV q 8 h), fentanyl (2 μg/kg/h IV), and carprofen (2.2 mg/kg SQ q 12 h) were started.

Fifteen hours later (day 1), the dog's care was transferred to the surgical service. On physical examination, severe soft tissue swelling persisted, consistent with cellulitis. Additionally, there was notable instability and crepitus at the distal tibia. Orthogonal radiographs of the distal left pelvic limb were repeated on day 1 and reviewed by the same radiologist (Figures 1C, D). Radiographic evidence of subcutaneous abscessation proximally from the crus and tarsus and distally to the metatarsal region was noted, and a Salter-Harris type I fracture of the distal tibial physis, likely pathologic and secondary to the previously suspected septic physitis, was diagnosed. Mild heterogeneity of the distal femur suggested possible concurrent hypertrophic osteodystrophy. Surgical reduction and fixation of the Salter-Harris fracture with concurrent tissue sample collection from the crus and tarsus for culture and susceptibility testing were recommended.

The dog was premedicated (acepromazine 0.05 mg/kg IV), and anesthesia was induced with methadone (0.1 mg/kg IV), midazolam (0.15 mg/kg IV), and propofol (6.0 mg/kg IV). Adequate anesthetic depth was maintained with isoflurane inhalant. A femoral and sciatic nerve block was administered with bupivacaine (2 mg/kg). Multimodal continuous rate infusion analgesia was provided with fentanyl (2.4 μg/kg/h) and ketamine (0.1 mg/kg/h). A medial approach was made to the left tarsus, and a large abscess was identified, which pocketed subperiosteally and extended into the distal tibial physis. The site was irrigated with sterile saline and necrotic tissue debrided. A tissue sample was collected from the subperiosteal abscess and the metaphyseal surface and submitted for aerobic and anaerobic culture (M040, Antech Diagnostics, Loveland, CO, USA). Intraoperatively, the physis was noted to be grossly gray and gelatinous. The fracture of the distal tibial physis was identified and manually reduced, then stabilized with two 1.6 mm Kirschner wires placed in cross-pin fashion. A 10 French, closed suction, Jackson-Pratt drain was placed in the subperiosteal space and exited through a new incision slightly proximal to the primary incision, prior to closure of the surgical wound.

**Figure 2:**
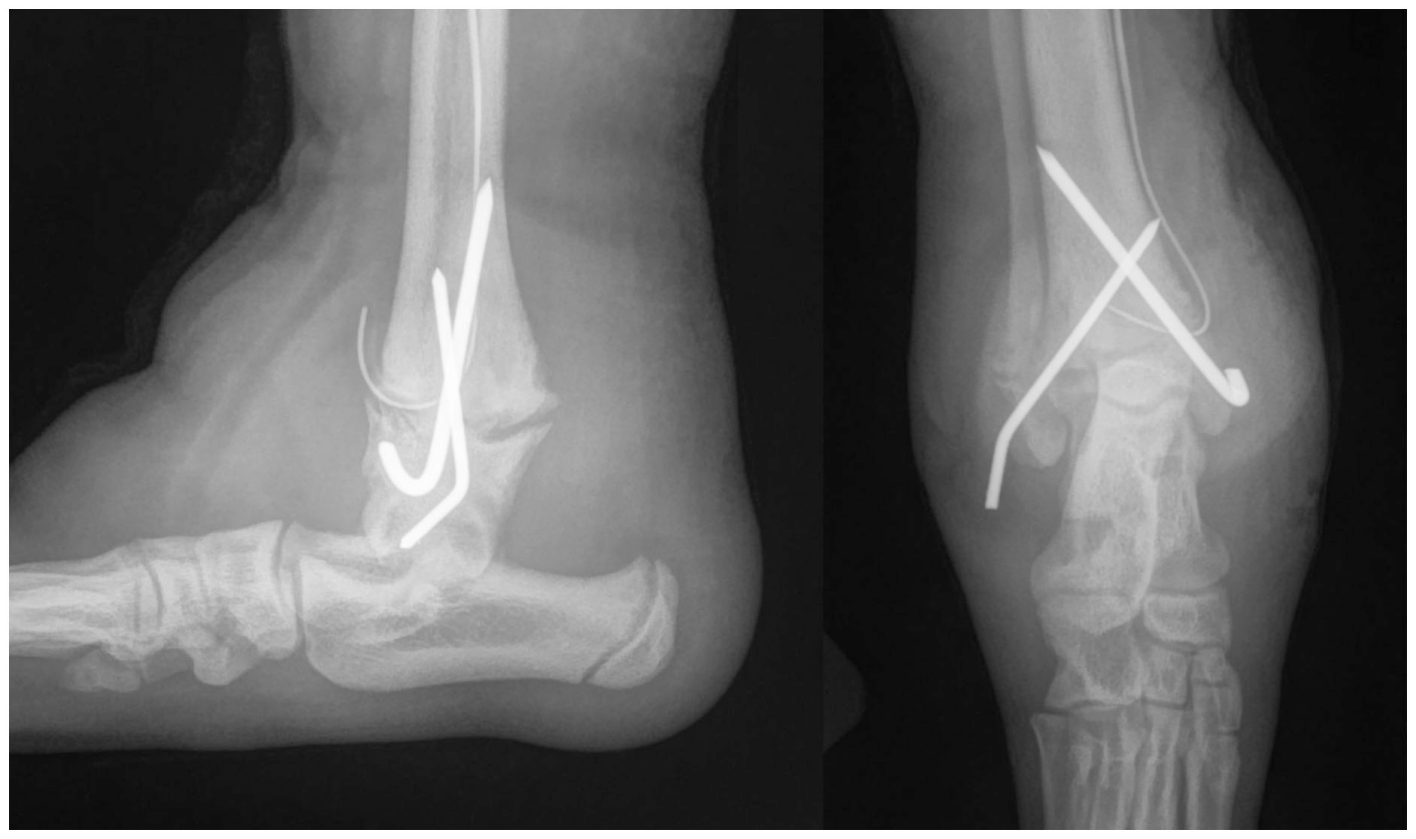
Two orthogonal radiographic projections of the left hock of the puppy described in Figure 1 postoperative to cross pin fixation of the Salter-Harris fracture and Jackson Pratt drain placement. Two 1.6 mm Kirschner wires were placed across the medial and lateral aspects of the physis in cross-pin fashion.

Prior to recovery from anesthesia, orthogonal radiographs of the distal tibia were performed, and appropriate alignment of the distal tibia was verified (Figure 2). Additionally, a newly documented focal lytic area of the distal femoral metaphysis raised concern for emerging sepsis causing polyostotic lesions. A lateral splint and modified Robert Jones bandage were applied, and the dog was recovered uneventfully from anesthesia. Postoperatively, the dog was administered intravenous fluid therapy (Plasmalyte A 60 mL/kg/day) and ketamine (0.1 mg/kg/h IV) for 4 h and was given carprofen (2.2 mg/kg SQ) and one additional dose of ampicillin/sulbactam (30 mg/kg IV). After 4 h, the dog was alert, responsive, normothermic (38.6 °C), and eating so gabapentin (7.5 mg/kg PO q 8–12 h), carprofen (1.9 mg/kg PO q 12 h), trazodone (3.7 mg/kg PO q 12 h), and amoxicillin/clavulanic acid (19 mg/kg PO q 12 h × 14 days) were prescribed to replace the injectable medications. Drain production prior to discharge was 0.8 ml/kg/h of serosanguinous fluid.

The dog was discharged from the hospital roughly 10 h after surgery with codeine (1 mg/kg PO q 8 h), gabapentin (7.5 mg/kg PO q 8–12 h), carprofen (1.9 mg/kg PO q 12 h), trazodone (3.7 mg/kg PO q 12 h), and amoxicillin/clavulanic acid (19 mg/kg PO q 12 h × 14 days). Additionally, the owners were instructed to aseptically empty and quantify the contents of the active suction reservoir bulb every 6 h.

On day 4, the dog was evaluated by the operating surgeon, and the drain was removed, and the bandage and splint were replaced. There was marked improvement in the swelling, ambulation with near full weight-bearing on the operated leg with the splint in place. The range of motion of the tarsus was normal and non-painful. Bandage changes were repeated on days 7, 11, and 16 with no evidence of complications or return of the edema, and the sutures were removed during the bandage change on day 16. The results of the culture and susceptibility testing identified growth of two colonies of *Pasteurella* spp., which was susceptible to amoxicillin/clavulanic acid, and the antibiotic was continued.

**Figure 3:**
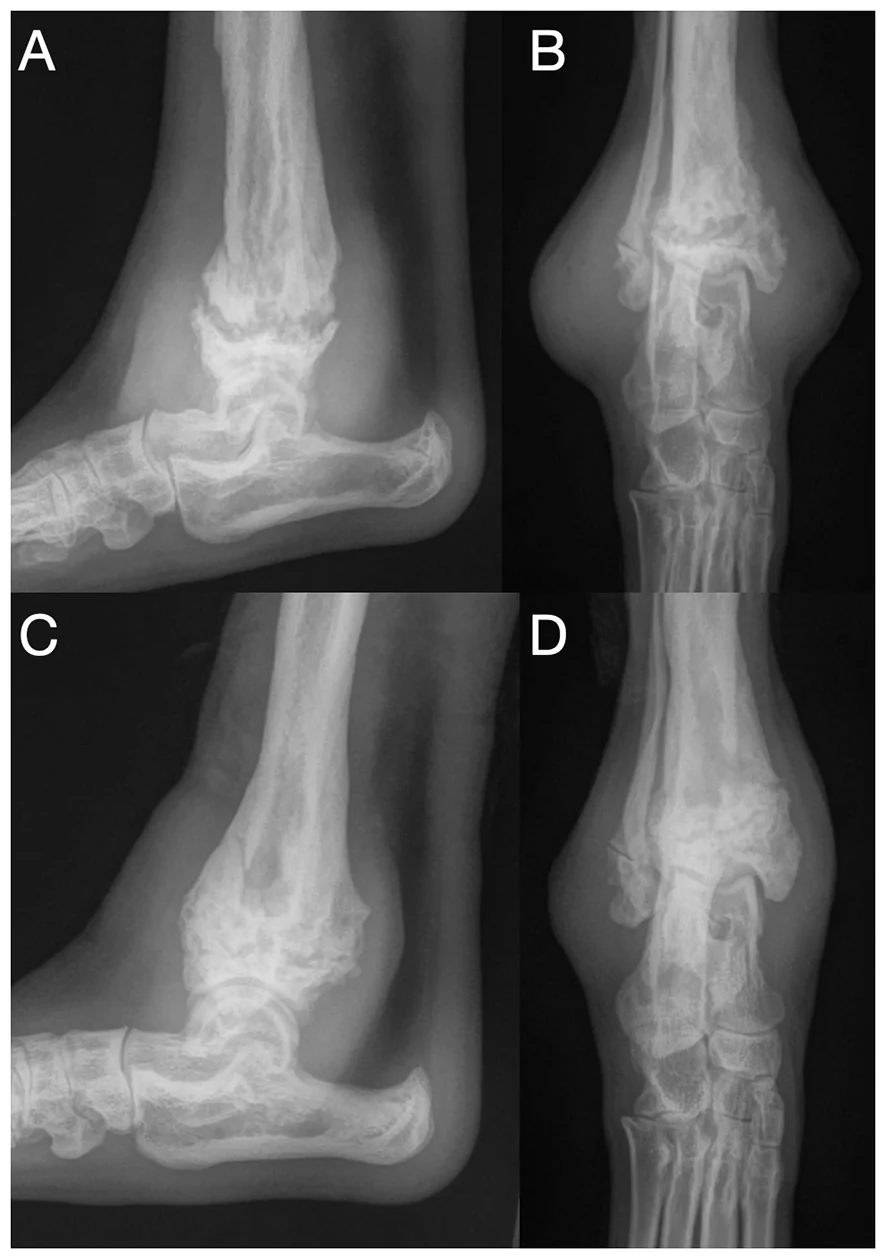
Two orthogonal radiographic projections of the left hock of the puppy described in Figure 1 after 30 **(A, B)** and 55 **(C, D)** postoperative days. **(A, B)** Two radiographs taken immediately following pin removal. There is evidence of chronic osteomyelitis and physitis of the left tibia with focal severe soft tissue swelling centered on the distal tibia and proximal tarsus. The physis is widened, irregular, and lucent with circumferential osseous proliferation and remodeling. **(C, D)** Progressive healing evident by progressive heterogenous remodeling and osseous unification of the left distal tibia in the recheck interim. Moderate regional soft tissue swelling is predominantly extracapsular and centered on the epiphysis and significantly improved from the previous time point.

On day 31, the dog (weight 17.7 kg) was evaluated by the operating surgeon. Physical examination revealed mild lateral swelling of the tarsus and minimal weight-bearing (4/5 lameness) on the operated leg without support of the splint. The dog was premedicated with acepromazine (0.02 mg/kg IM), trazodone (5.6 mg/kg PO), hydromorphone (0.1 mg/kg IV), dexmedetomidine (4 μcg/kg IV), and anesthesia was induced with propofol (0.6 mg/kg IV) prior to performing two orthogonal radiographic projections of the distal tibia. There was a reduction in soft tissue swelling and sclerosis compared to the immediate postoperative period, though focal swelling persisted around the distal tibia and proximal tarsus. The distal tibial physis remained widened, irregular, and lucent, with circumferential osseous proliferation and remodeling, indicating suspected ongoing chronic osteomyelitis and physitis (Figure 3). The surgical scar was aseptically prepared, and the pins were removed through a small incision. The pins and a cotton swab inoculated from the surgical wound were submitted for aerobic and anaerobic culture and susceptibility (M040, Antech Diagnostics). Bupivacaine (1.5 mg/kg) was infiltrated into each site. A modified Robert Jones bandage was placed prior to uneventful recovery from anesthesia and discharge from the hospital.

On day 37, the dog was evaluated by the operating surgeon. The dog was ambulating well with the bandage in place, and there was no significant swelling of the distal tibia with normal and comfortable tarsal range of motion. The splint and modified Robert Jones bandage were replaced. No organisms were identified with the culture and susceptibility test, and the amoxicillin/clavulanic acid was prescribed for an additional 6 weeks (discontinue on day 73), with a dose increase (20 mg/kg PO q 12 h) for the final 3 weeks due to the puppy's growth.

On day 45, the dog was presented to the operating surgeon for evaluation and removal of the bandage. Physical examination of the operated limb found no swelling or wounds at any level, and the range of motion of the tarsus was comfortable on manipulation. The bandage was not replaced.

On day 57, the dog was evaluated for the final time. Physical examination was consistent with appropriate ambulation in all four limbs with no lameness noted in the operated limb. Radiographic examination of the distal tibia was consistent with progressive osteogenesis at the site of the left distal tibial physis, with radiographic evidence of apparent but improved chronic osteomyelitis and regional soft tissue swelling (Figure 3). At the time of the final examination, the dog was 6 months old, and there was no evidence of complete or partial premature closer of the distal tibial physis.

## Discussion

The definitive mechanism of Salter-Harris type I fracture in this puppy remains unclear; however, diagnostic imaging and surgical findings support that the Salter-Harris Type I fracture was secondary to suspected septic physitis. In the absence of a known penetrating injury, hematogenous dissemination was hypothesized to be the primary route of infection, potentially originating from transient bacteremia associated with the dog's normal flora. A similar mechanism has been documented in large animals with hematogenous spread of bacteria in juvenile equines leading to septic physitis with fracture secondary to severe infection and bony inflammation of the affected physis (8). While it remains unclear why this disease process is seen primarily in large animals (potentially due to their reduced capacity to bear weight on three limbs), our case demonstrates that the same mechanism may exist in small animals, and a pathologic appendicular fracture can be a potential sequela of septic physitis in dogs. The physis is the weakest region of an appendicular bone of a growing animal (11). Compromise of the metaphysis from hematogenous osteomyelitis combined with mechanical force is a proposed mechanism for pathologic Salter-Harris Type I fracture in this case. Additionally, we demonstrated that rapid detection of pathologic fracture in cases of septic physitis and aggressive therapy led to a good outcome. This is unlike cases documented in foals with fractures secondary to hematogenous osteomyelitis and in cattle where surgical internal fixation might not be a viable option (9, 10).

Salter-Harris fracture secondary to hematogenous septic physitis has been reported in foals. However, unlike in our case, the septic physitis in foals is caused by *E. coli, Salmonella* spp.*, Actinobacillus* spp.*, Klebsiella* spp.*, Staphylococcus* spp.*, Streptococcus* spp., and *Rhodococcus equi* (7, 8). In foals, hematogenous bacteria are predominantly from failure of passive transfer, gastrointestinal, umbilical or respiratory infections (7, 8). In this dog, *Pasteurella multocida*, a ubiquitous gram-negative, facultative anaerobe, which is commonly found in the oropharynx of cats and dogs, was isolated (12). Hematogenous spread of bacteria may subsequently occur, resulting in joint sepsis, osteomyelitis, or subcutaneous abscessation (6). Through hematogenous spread, bacteria may colonize in areas of endochondral ossification within the metaphysis and epiphysis of long bones and vertebral bodies. In juvenile dogs with open physes, the capillaries within these regions are fenestrated, providing an environment for bacteria to enter the metaphysis and cause subsequent infection (6). This is hypothesized to be the mechanism by which septic physitis develops in juvenile small animals with the carpus and stifle being overrepresented (6). It is unclear why it occurred in the tarsus of the puppy described in this case. Since there was no known history or evidence of direct penetrating injury, such as bites wounds, we suspect that the infection caused by *Pasteurella multocida*, which is often found in canine oral cavities, resulted from hematogenous bacterial spread from minor asymptomatic or transient oral infection by normal flora (1–4, 12).

While *Pasteurella* multocida was isolated in this case it, it is possible that the colony growth represented contamination rather than true infection. However, the samples were collected aseptically during a surgical procedure where aseptic technique was maintained, and the culture and susceptibility testing was performed in a veterinary diagnostic laboratory following optimal sample processing guidelines. Due to the clinical signs, radiographic findings consistent with septic physitis, and intraoperative visualization of subperiosteal abscessation, it is presumed that the growth of *Pasteurella* spp. was not a contamination, though bone culture and histology would have provided a more definitive diagnosis in this case.

Rigid fixation of Salter-Harris fractures is recommended as soon as possible to facilitate anatomic reduction and minimize complications associated with delayed intervention, as the high healing potential of growing puppies can result in rapid callus formation that might complicate anatomic fracture reduction (13). However, that plan was complicated by subperiosteal abscess formation and osteomyelitis at the site. Although external coaptation, transfixation pin casting, or external skeletal fixation could have been considered, it was hypothesized that rigid internal fixation offered the greatest likelihood of achieving anatomic reduction and stable fixation of the physeal fracture despite the inherent risk of implant contamination and potential contamination of the adjacent tibiotarsal joint during pin placement. Despite the risks associated with using implants in an infected surgical site, rapid surgical intervention and initiation of intravenous beta lactam antibiotics, which were appropriate for *Pasteurella* spp., likely contributed to the positive clinical response in this case. The dog responded well to treatment with progressive improvement in limb function and full resolution of pain and lameness. The dog's recovery, with no residual lameness and full tarsal range of motion, supports a favorable overall outcome with this treatment approach, though radiographic examination after skeletal maturity is unavailable to check, so information regarding potential tibial shortening or deformity that could indicate premature closure of the growth plate despite surgical reduction is unavailable.

Treatment recommendations for septic physitis in foals includes 4–6 weeks of systemic antimicrobial therapy, with duration guided by resolution of clinical signs and serial diagnostic imaging results (7, 8). Since evidence regarding optimal systemic antibiotic therapy for canine septic physitis is lacking, especially with the addition of surgical implants at the site of the infection, the surgeon followed the recommendations for foals. However, due to the perceived increased risk of poor bacterial clearance at surgical sites involving implants, the surgeon prescribed the antibiotics for the duration of the implant presence before beginning the 4–6-week prescription, resulting in 72 total days of antibiotic therapy. While this puppy improved clinically and radiographically with this treatment plan, further study is needed to guide optimal duration of antibiotic therapy for suspected physitis with or without an associated Salter-Harris fracture.

Although the rapid progression of the fracture was theorized to be secondary to suspected septic physitis, the presence of a pre-existing, non-displaced fracture at the time of initial radiographic evaluation cannot be definitively excluded. Musculoskeletal ultrasound of the affected joint could have been used to further evaluate for the presence of a non-displaced fracture following radiographic identification of widening of the growth plate, although sensitivity and specificity of musculoskeletal ultrasound is not recommended for definitive diagnosis of Salter-Harris type I fractures (14). It is possible that subsequent displacement, accompanied by increased inflammation and clinical deterioration, prompted repeat imaging, which ultimately facilitated diagnosis of the fracture. Advanced imaging modalities, such as computed tomography (CT) or magnetic resonance imaging (MRI), may have provided additional diagnostic clarity. Though there was evidence of sclerosis suggestive of premature closure of the affected physis, long-term radiographic follow-up was not available. Therefore, the possible impacts of premature physeal closure, potentially resulting in limb shortening or angular deformity, could not be assessed, despite aggressive and rapid surgical reduction and fixation. The dog was re-presented to the referral hospital approximately one year later for unrelated, self-limiting gastrointestinal signs. At that time, no lameness or mobility deficits were reported or observed.

## Conclusion

The Salter–Harris type I fracture in this puppy, likely secondary to suspected septic physitis, highlights that young dogs are susceptible to osteomyelitis involving the diaphysis, metaphysis, or physis, and that severe infection can rapidly weaken the growth plate to the point of pathologic fracture through the physis. Due to these findings, septic physitis should be considered a differential when puppies are presented with idiopathic physeal fractures.

## Data Availability

The original contributions presented in the study are included in the article/supplementary material, further inquiries can be directed to the corresponding author.

## References

[1] Gieling F Peters S Erichsen C Richards RG Zeiter S Moriarty TF. Bacterial osteomyelitis in veterinary orthopedics: pathophysiology, clinical presentation and advances in treatment across multiple species. Vet J. (2019) 250:44–54. doi: 10.1016/j.tvjl.2019.06.003 31383419

[2] Arons MS Fernando L Polayes IM. Pasteurella multocida—the major cause of hand infections following domestic animal bites. J Hand Surg Am. (1982) 7:47–52. doi: 10.1016/S0363-5023(82)80013-0 7061808

[3] González-Martín M Silva V Poeta P Corbera JA Tejedor-Junco MT. Microbiological aspects of osteomyelitis in veterinary medicine: drawing parallels to the infection in human medicine. Vet Q. (2022) 42:1–11. doi: 10.1080/01652176.2021.2022244 34936853 PMC8725753

[4] Johnson KA. Osteomyelitis in dogs and cats. J Am Vet Med Assoc. (1994) 204:1882–7. doi: 10.2460/javma.1994.204.12.1882 8077129

[5] Salter RB Harris WR. Injuries involving the epiphyseal plate. J Bone Joint Surg. (1963) 45:587–622. doi: 10.2106/00004623-196345030-00019

[6] Ruocco NA 3rd Luedke LK Fortier LA Ducharme NG Reesink HL. Rhodococcus equi joint sepsis and osteomyelitis is associated with a grave prognosis in foals. Front Vet Sci. (2020) 6:503. doi: 10.3389/fvets.2019.00503 31993449 PMC6971166

[7] Verschooten F Vermeiren D Devriese L. Bone infection in the bovine appendicular skeleton: a clinical, radiographic, and experimental study. Vet Radiol Ultrasound. (2000) 41:250–60. doi: 10.1111/j.1740-8261.2000.tb01488.x 10850877

[8] Glass K Watts AE. Septic arthritis, physitis, and osteomyelitis in foals. Vet Clin North Am Equine Pract. (2017) 33:299–314. doi: 10.1016/j.cveq.2017.03.002 28687092

[9] Hardy J. Etiology, diagnosis, and treatment of septic arthritis, osteitis, and osteomyelitis in foals. Clin Tech Equine Pract. (2006) 5:309–17. doi: 10.1053/j.ctep.2006.09.005

[10] Leonhardt LP Pervez A Tang W. Pasteurella multocida bacteremia and septic arthritis in a patient with non-bite animal exposure. Cureus. (2021) 13:*e*14162. doi: 10.7759/cureus.14162 33936874 PMC8079206

[11] Sukhiani HR Holmberg DL. Ex vivo biomechanical comparison of pin fixation techniques for canine distal femoral physeal fractures. Vet Surg. (1997) 26, 398-407. doi: 10.1111/j.1532-950X.1997.tb01700.x 9381666

[12] Beatty E Archambault P. BET 1: can Salter-Harris type I fractures be diagnosed by ultrasound? Emerg Med J. (2018) 35:335–6. doi: 10.1136/emermed-2018-207686.1 29674383

[13] Wei SQ Guo C Lu ZJ Lin FX. From acute infection to chronic osteoimmune remodeling: immunopathogenesis and precision immunotherapeutic strategies in osteomyelitis. Int Immunopharmacol. (2026) 181:116669. doi: 10.1016/j.intimp.2026.116669 42061139

[14] Arnold SR Giordano V Obremskey WT Schwarz EM Smeltzer MS Veis DJ . Osteomyelitis. Nat Rev Dis Primers. (2026) 12:33. doi: 10.1038/s41572-026-00707-9 42209515

